# Lockdown and licensed premises: COVID‐19 lessons for alcohol policy

**DOI:** 10.1111/dar.13413

**Published:** 2021-12-13

**Authors:** Niamh Fitzgerald, Francesco Manca, Isabelle Uny, Jack Gregor Martin, Rachel O'Donnell, Allison Ford, Amelie Begley, Martine Stead, Jim Lewsey

**Affiliations:** ^1^ Institute for Social Marketing and Health University of Stirling Stirling UK; ^2^ SPECTRUM Consortium Edinburgh UK; ^3^ Institute of Health and Wellbeing University of Glasgow Glasgow UK

**Keywords:** COVID‐19, alcohol policy, alcohol availability, ambulance, licensing

## Abstract

**Introduction:**

The COVID‐19 pandemic necessitated unprecedented changes in alcohol availability, including closures, curfews and restrictions. We draw on new data from three UK studies exploring these issues to identify implications for premises licensing and wider policy.

**Methods:**

(i) Semi‐structured interviews (*n* = 17) with licensing stakeholders in Scotland and England reporting how COVID‐19 has reshaped local licensing and alcohol‐related harms; (ii) semi‐structured interviews (*n* = 15) with ambulance clinicians reporting experiences with alcohol during the pandemic; and (iii) descriptive and time series analyses of alcohol‐related ambulance callouts in Scotland before and during the first UK lockdown (1 January 2019 to 30 June 2020).

**Results:**

COVID‐19 restrictions (closures, curfews) affected on‐trade premises only and licensing stakeholders highlighted the relaxation of some laws (e.g. on takeaway alcohol) and a rise in home drinking as having long‐term risks for public health. Ambulance clinicians described a welcome break from pre‐pandemic mass public intoxication and huge reductions in alcohol‐related callouts at night‐time. They also highlighted potential long‐term risks of increased home drinking. The national lockdown was associated with an absolute fall of 2.14 percentage points [95% confidence interval (CI) −3.54, −0.74; *P* = 0.003] in alcohol‐related callouts as a percentage of total callouts, followed by a daily increase of +0.03% (95% CI 0.010, 0.05; *P* = 0.004).

**Discussion and Conclusions:**

COVID‐19 gave rise to both restrictions on premises and relaxations of licensing, with initial reductions in alcohol‐related ambulance callouts, a rise in home drinking and diverse impacts on businesses. Policies which may protect on‐trade businesses, while reshaping the night‐time economy away from alcohol‐related harms, could offer a ‘win–win’ for policymakers and health advocates.

## Introduction

The COVID‐19 pandemic necessitated unprecedented changes in alcohol availability with closures, curfews and operating restrictions in many countries. Complete sale bans were introduced in several countries [1, 2], whereas in others, including the UK and Australia, alcohol retailers were designated as essential services and permitted to trade throughout the pandemic [3]. Shops and online retailers, including supermarkets, are likely to have benefitted significantly [3, 4], where they stayed open while bars were closed.

Other pandemic control measures also have implications for where, when and how much alcohol is drunk. It may be ‘easier’ to drink alcohol where people work more from home with less need to drive and lower visibility [5]. Greater consumption or alcohol problems may also be driven by increased caring responsibilities, stress levels, bereavement, isolation, job insecurity and poverty with reduced access to services and social supports, particularly affecting women, ethnic minority and economically disadvantaged groups [5, 6]. Accurately assessing and appropriately responding to the above changes is not easy. In the UK, overall alcohol sales fell during the first national lockdown (driven by falls in on‐trade sales, especially beer, while premises were closed), but then largely recovered [7]. UK survey data from early in the pandemic indicates that high‐risk drinking may have increased, more so in women and disadvantaged groups, while affluent groups were more likely to report attempts to cut down their consumption, raising concerns about exacerbations of existing inequalities [8, 9]. Internationally, findings are diverse with some studies reporting stability [10], others increases in consumption and harms [11, 12] or decreases [13, 14], but with unequal distribution of changes in population subgroups found in these and other studies [10, 15, 16, 17]. With the exception of a survey in the Americas, which found that increases in heavy episodic drinking were more common in men [14], many studies have raised concerns about disproportionate increases in women's drinking, perhaps due to stress [18, 19, 20].

Alongside changes in alcohol consumption, a general decline in healthcare utilisation for non‐COVID‐19 issues was observed early in the pandemic, with decreases across wide ranging conditions [21, 22, 23, 24]. A US study found a 31% decrease in ambulance responses for April 2020 compared to the previous year [25]. Fear of infection and a desire to avoid burdening health services may explain these falls; though health behaviours, including alcohol consumption may also be a factor. In Canada, alcohol‐related visits at accident and emergency departments decreased at the beginning of the pandemic but to a lesser extent than other visits [26]. In New York City, during the initial COVID‐19 peak in spring 2020, hospital visits for alcohol withdrawal increased while those for alcohol use decreased [27]. To date, to the best of our knowledge, the impact of COVID‐19 on alcohol‐related ambulance callouts has not been reported.

Much UK public discourse has centred on hardships for hospitality businesses arising from public health measures with surprisingly little discussion of reduced pressures on emergency services [28, 29]. The burden of alcohol on health services is well documented, including recent work estimating that 16.2% of all ambulance call‐outs in Scotland are alcohol‐related [30]. This discourse contrasts with examples elsewhere of governments placing or maintaining restrictions on alcohol explicitly to reduce this burden on front‐line services [31, 32]. Shifts to home drinking may lead to increases in consumption, gender‐based violence or risks for children [5, 33], including neglect or modelling of parental drinking [34, 35]. Much is still to be understood about the impact of COVID‐19 on alcohol consumption, harms and services: the diversity in methods, local experiences and control measures and the time periods of each study, makes interpretation difficult and a nuanced approach is necessary.

Meanwhile government policy has moved on apace in many countries to considering post‐pandemic recovery. While some public health experts have drawn attention to the need for stronger alcohol controls to protect health services and public health [4, 36], there are signs that an emphasis on protecting business recovery may make politicians reluctant to act [37]. Consideration of the balance of regulation affecting on and off trade premises will be critical, having perhaps been neglected in the past [3] and there is an urgent need for evidence to inform policy deliberations. Existing studies which can ‘pivot’ towards COVID‐19 can provide timely, relevant data.

In this paper, we: (i) review primary qualitative data from interviews which took place during the pandemic for two separate studies not originally focused on COVID‐19; and (ii) analyse secondary data on alcohol‐related ambulance call‐outs during the first UK national lockdown. Using this data, we seek to inform discussions of the following three questions:

1. How might the pandemic have reshaped regulation of alcohol sales via the local premises licensing system in England and Scotland?
2. What are ambulance clinicians' experiences and views of alcohol‐related ambulance callouts during the pandemic?
3. How did alcohol‐related ambulance callouts in Scotland change in volume and timing during the first UK national lockdown compared to non‐alcohol callouts?

Together, these sources can contribute to an understanding of the potential implications of changes in the alcohol regulatory environment and related behaviours during the pandemic, for public health and emergency service utilisation in the short and medium term.

## Methods

### Overview and ethics

First, we report in‐depth interview data from a study on the role of public health in licensing [38] in which professional stakeholders interviewed in late 2020 discussed how COVID‐19 had affected licensing issues. Second, we report ambulance clinicians' experiences of alcohol‐related callouts during the pandemic, from in‐depth interviews also conducted as part of a broader study [39]. Ethical approval was granted by the University of Stirling National Health Service, Invasive or Clinical Research ethics committee for both studies (NICR 16/17‐064/064A and NICR 19/20056) and full informed consent obtained from participants. Finally, we present descriptive statistics for overall and alcohol‐related ambulance callouts in Scotland before and after the national UK lockdown. No ethical approval was required for the secondary analysis of non‐identifiable callout data, but Research and Development governance approval was obtained from the Scottish Ambulance Service.

### Context

**Figure 1:**
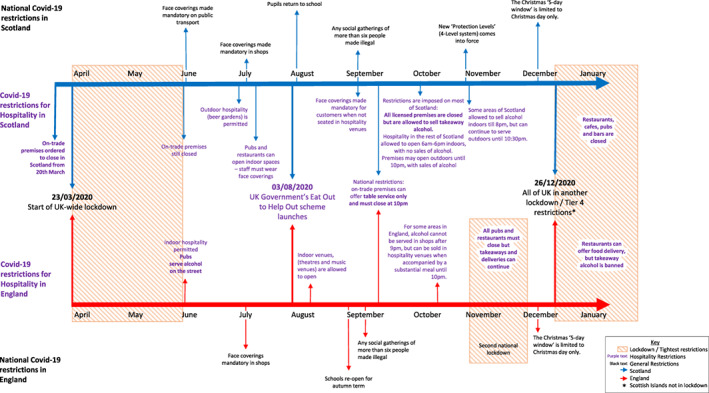
Map of COVID‐19 restrictions in Scotland and England.

In response to the pandemic, the UK Government, in tandem with the Scottish Government, initiated a national lockdown requiring all ‘on‐licence’ premises (where alcohol is sold for consumption on the premises), including bars, restaurants and nightclubs, to stop trading from 20 March 2020. In Scotland, such premises remained fully closed until 19 June, when they were permitted to re‐open to serve alcohol in outdoor spaces (e.g. beer gardens) only, with indoor spaces opening in Scotland from 15 July. From early July in England, premises gradually re‐opened with social distancing and other requirements in place, albeit implemented to varying degrees [40]. Prevailing restrictions on trading were stepped up or down throughout 2020 and the first half of 2021, varying geographically within and between Scotland and England. ‘Off‐licence premises’ (shops licensed to sell alcohol) were permitted to open throughout the pandemic period. To summarise, the biggest changes in alcohol availability at various times were: (i) complete closures of on‐licensed premises; (ii) rolling closures of on‐licence premises in some areas, with imposed early closing times when open; and/or (iii) a ban on sales of alcohol indoors (in Scotland only). COVID‐19 restrictions were generally imposed by the national (UK) or devolved (Scottish) Governments, but there was some local variation due to the localised system of licensing of premises that exists in the UK, see [38, 41]. A detailed timeline of changes in Scotland and England is provided in Figure 1.

### Study 1: In‐depth interviews from the ‘Evaluating the impact of alcohol licensing in England and Scotland’ (ExILEnS) study

*Sampling*: As part of the wider ExILEnS study [38], we recruited 20 public health teams covering 14 English and six Scottish areas, varied in rurality and region, where the public health team was actively engaged in alcohol licensing. Purposive sampling of individual stakeholders for in‐depth telephone interviews focused on diversity in terms of location (Scotland vs. England) and remit of interviewees (public health, licensing staff, police, local politicians and licensing lawyers). *Recruitment and consent*: Potential participants were identified via professionals who took part in initial site visits and/or interviews, with researchers following up with nominated individuals. Each was provided with an information sheet and gave written or audio‐recorded informed consent. *Final sample and data collection*: Of 53 interviews conducted, 17 were conducted during the COVID‐19 pandemic between August and October 2020 (see Figure 1). These 17 interviews form the dataset reported here. All were audio‐recorded. Bespoke interview topic guides were developed for interviewees with different remits and included questions relevant to licensing on public health team activities, outcomes and alcohol‐related harms. The same topic guides were used pre‐ and during COVID‐19. The 17 interviews (averaging 74 min) were not specifically focused on COVID‐19, but 15 participants spontaneously raised the topic. Table 1 outlines the profile of these interviewees. *Analysis*: Interviews were professionally transcribed, then anonymised and imported into NVivo 12 for reflexive thematic analysis. This approach was used given its focus on fluid coding processes and reflection on/engagement with the data. RO coded all transcripts against a set of categories created using deductive (reviewing research questions and topic guides) and inductive approaches (reading transcripts), to describe potentially important features in the data. One category was ‘COVID‐19’. After initial coding, all COVID‐19 extracts from the 15 interviews were reviewed in detail by NF in discussion with RO, to identify the range and diversity of responses in relation to this topic, which formed the dataset for this paper. NF wrote up the findings, which were reviewed and refined in discussion with RO, with any discrepancies regarding interpretation resolved between both authors.

**Table 1:** Study 1 profile of interviewees providing data for this paper

Interview no.	Country	Stakeholder group	Gender	Experience (years)	Interview date	Duration (min)
46	England	Public Health	Female	3	4 September 2020	58
53	England	Public Health	Female	20	17 September 2020	78
43	England	Licensing Team	Male	17	21 August 2020	85
44	England	Licensing Team	Female	17	26 August 2020	59
38	Scotland	Licensing Team	Male	10	12 August 2020	72
42	England	Elected Representative	Male	3	20 August 2020	72
40	England	Elected Representative	Male	2	14 August 2020	73
45	England	Elected Representative	Female	14	03 September 2020	90
48	Scotland	Elected Representative	Male	11	09 September 2020	93
50	Scotland	Elected Representative	Female	13	15 September 2020	60
54	England	Licensing Lawyer	Female	10	28 September 2020	53
39	Scotland	Licensing Lawyer	Male	24	13 August 2020	73
49	Scotland	Licensing Lawyer	Female	7	14 September 2020	82
47	England	Licensing Police	Male	2	09 September 2020	91
41	Scotland	Licensing Police	Female	17	20 August 2020	72

### Study 2: In‐depth interviews from ‘Impact of minimum pricing for alcohol on ambulance callouts in Scotland’ (IMPAACT) study

Study 2 consisted of in‐depth qualitative interviews as part of a wider study which started in May 2019 and will finish at the end of 2021 and which focused on the impact of alcohol and of minimum unit pricing of alcohol, on ambulance callouts and ambulance service provision. *Sampling*: We recruited 27 ambulance clinicians working in frontline practice for the Scottish Ambulance Service (SAS), with diversity in terms of experience, seniority, gender and role (e.g. paramedics, paramedic technicians) as well as geographic variation across regions. *Recruitment and consent*: Eligible clinicians were invited to express interest in taking part via an email from SAS managers and then through a blanket email to all frontline clinicians in SAS. Participants were provided with an information sheet and gave written or audio‐recorded informed consent. *Final sample and data collection*: From those who expressed interest (*n* = 118), 27 were selected in accordance with our sampling strategy and took part in telephone interviews, of which 15 were conducted during the COVID‐19 pandemic. Although the interview questions were mainly on the impact of alcohol on SAS, 13 participants spontaneously raised the issue of changes in alcohol‐related harms during the pandemic (see Table 2). They were prompted further on the topic by the researcher. These discussions form the dataset for this paper. Interviews were audio‐recorded. *Analysis*: With participant permission, interview recordings were professionally transcribed, anonymised and imported into NVivo 12 for analysis. Three members of the research team (IU/JM/AF) coded the transcripts against a set of categories created using deductive (reviewing research questions and topic guides) and inductive approaches (reading transcripts). After the initial coding, NF, JM and IU discussed the emerging themes and extracts from the transcripts which related to COVID‐19 in detail. This supported the writing of the first draft of findings by NF, which JM and IU reviewed before submission.

**Table 2:** Study 2 profile of interviewees providing data for this paper

Interview no.	Region	Job title	Gender	Experience (years)	Interview date	Duration (min)	COVID‐19 topic raised by?
14	East	Technician and trainee paramedic	Male	4	23 April 2020	106	Interviewee
15	North	Technician	Female	13	29 April 2020	90	Interviewee
16	West	Technician	Male	4	01 May 2020	65	Interviewee
22	North	Ambulance technician and other	Male	11	12 August 2020	35	Interviewer
23	West/North	Air crew	Male	16	12 September 2020	81	Interviewer
17	West	Paramedic	Male	11	11 December 2020	77	Interviewee
26	East	Paramedic	Male	36	22 January 2021	62	Interviewee
27	East	Advanced paramedic	Female	25	25 January 2021	81	Interviewee
24	West	Ambulance technician	Male	4	14 December 2020	85	Interviewer
18	East	Paramedic	Male	32	17 November 2020	101	Interviewee
25	West	Other	Male	28	20 January 2021	45	Interviewee
19	West	Ambulance technician	Female	3	27 November 2020	91	Interviewee

### Study 3: Lockdown and licensed premises

Study 3 utilised electronic patient record data from SAS to describe trends in alcohol‐related ambulance callouts in Scotland between 1 January 2018 and 30 June 2020, before and during the first UK‐wide COVID‐19 lockdown when all on‐licence premises were closed. Alcohol‐related ambulance callouts were defined as those identified using an algorithm that makes use of free text notes completed by ambulance staff in electronic patient records for each callout as well as an alcohol ‘flag’—a field in the electronic patient record allowing ambulance clinicians to indicate if a callout is alcohol‐related. The algorithm was validated and performed well with 98% accuracy [30]. Our analyses utilised both a descriptive and an inferential approach. First, we created graphs and tables describing ambulance callouts over time, alcohol‐related or not. To facilitate comparison, we also computed alcohol‐related callouts per 100 000 residents using mid‐year population estimates [42]. Then, we conducted an interrupted time series analysis [43] on alcohol‐related callouts as a percentage of total callouts. Specifically, a seasonal autoregressive integrated moving average analysis was performed to take into account of seasonal variations in the callout trends and a change in level and of slope associated with lockdown was tested for. We analysed the proportion rather than the count of alcohol‐related callouts to isolate the effect of lockdown on alcohol alone, removing any effect of a general decline in health care (and ambulance service) demand during the first waves of the pandemic.

We discuss the findings of each of the studies in turn below.

## Results

Findings across these studies provide evidence of profound changes due to COVID‐19 and related public health measures that are relevant to alcohol policy, including the practices and experiences of public sector professionals, licensed businesses and members of the public.

### Findings from the ExILEnS study of public health involvement in licensing

Key issues identified from this analysis included reductions in stakeholders' focus on licensing, concerns about alcohol‐related harms associated with relaxation of licensing rules and observations on the impact of restrictions on different licensed businesses.

Licensing stakeholders reported that COVID‐19 had ‘*changed the whole face of licensing*’ and gave specific examples relating to changed priorities and ways of working. Some public health actors had to withdraw from their work on alcohol licensing to focus on pandemic‐related work, halting partnership work with other licensing stakeholders. One public health actor raised concerns on this basis, suggesting that new licensing applications/proposed variations to existing licenses might not be given the same level of scrutiny from a public health perspective. In contrast, other partnership working, such as between the police and environmental health officers and licence holders and elected members, was strengthened because of opportunities to work more closely together on COVID‐19‐related matters. Second, several stakeholders expressed concern about relaxation of licensing regulations, which they feared might not fully reversed. A national decision was taken in England to permit licensed premises forced to close during the lockdown to sell takeaway alcohol, resulting in ‘*people wandering around the street with, you know, plastic pint pots, which is what they're allowed to do now*’ (F, Elected Member England). This was reportedly done without consultation and experienced as ‘*pulling the rug out from under*’ (undermining) local licensing stakeholders. As businesses reopened following the first UK lockdown, interviewees reported that multiple licences were granted to permit the sale of alcohol for consumption in areas outside premises. Licensing team members explained that the volume of such applications meant they were unable to visit premises prior to applications being considered as they normally would do. A public health interviewee suggested that some premises would ‘*sneak in an hour change*’ (a request for additional hours of trading), as part of these applications. Another raised concern about expansion in availability through outdoor drinking when premises re‐opened because ‘*especially just now…everybody's needing to turn everything in to a beer garden just to keep the business going, you know, with social distancing etc*.’ (O, Police, Scotland).

Finally, interviewees reported unevenly distributed impacts of COVID‐19 on businesses, noting that some were more vulnerable to collapse, including ‘*small corner pubs*’, nightclubs and live music venues. Those able to provide online or home delivery sales were well‐positioned to benefit from the pandemic, but stakeholders were concerned about whether age verification procedures would be properly implemented, with delivery drivers having to maintain social distancing. Nightclubs were required to remain closed until the second half of 2021, but many ‘hybrid’ late‐night venues with similar characteristics to nightclubs were permitted to open.

### Findings from the IMPAACT study of alcohol's impact on the ambulance service

Ambulance clinicians reported ‘*huge differences*’ as the number of callouts relating to alcohol they attended late at night had ‘*plummeted*’, when premises were closed completely or under curfew, although there were some increases in domestic callouts and concerns raised about home drinking.

Closures and other restrictions affecting pubs and nightclubs meant ‘*nowhere near the same amount of public intoxication or mass intoxication… there's been much less in the way of assaults that involve alcohol, unconscious people outside that involve alcohol, falls that involve alcohol, all these things we've noticed a massive drop in*’ (Interviewee #24). Another welcomed this change, explaining:

‘*It's so nice to go to work on a Friday night knowing that you don't have to go into pubs and clubs… it's made a huge difference. And although you know you will get occasions, you will get parties, you will get illicit parties or you will get people still drinking, but you don't get that whole war, you know battlefield environment…*’ (#26).

Others reported that they were no longer dealing with ‘*drunken idiots*’ and therefore ‘*going to genuine calls*’ (#27). This contrasted with the initial period of time when pubs/bars reopened after the first national lockdown, which was described as ‘*pandemonium*’ (#25). One paramedic reported that the reduction in callouts experienced when premises were closed in the first lockdown (March to June 2020) was more pronounced than in the second in early 2021.

Alongside this reduction, domestic alcohol‐related incident numbers appeared to have gone up, though ‘*not a massive increase*’ (#14). Tensions or domestic violence in homes were felt to be exacerbated by couples being forced to spend more time at home together. Second, the ease of internet purchases and supermarket home deliveries of alcohol meant people did not need to leave their houses to access alcohol. Third, people with prior alcohol dependence were reported to be ‘*now struggling because they have nothing else to do*’ (#27). Others reported that ‘[people with alcohol issues] *now can't go out and they sit in the house all day drinking, and they drink and drink and drink and drink and drink*’ and added their view that isolation from friends affects the mental health of these patients.

While some ambulance clinicians expressed a hope that people might be less inclined to call an ambulance for minor issues post‐pandemic, others described fears about longer‐term consequences arising from new alcohol consumption habits formed during the pandemic that might persist.

*‘… alcohol intake is definitely increased in the house. My thoughts on when this is all finished, the people that used to go socializing, might not… I still think the younger generation will go back out to the nightclubs and the public houses and that, probably forty year old people upwards…I think they might get used to being in the house drinking, why get dressed and go out, when I can just sit here and watch TV and do what I've been doing for the last seven months*’ (#18)

*‘All these people can't stay at home drinking without a consequence at some point*.’ (#23)

### Findings from the study of alcohol‐related ambulance‐callouts during lockdown

**Figure 2:**
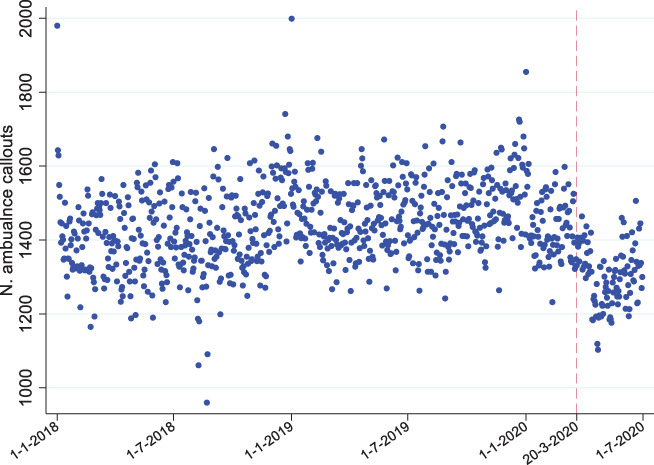
Total ambulance callouts in Scotland. Each dot = daily ambulance callouts. Dashed red line indicates the date of the order to close all hospitality venues (including pubs). Outliers correspond to public holidays, including New Year's Eve.

**Figure 3:**
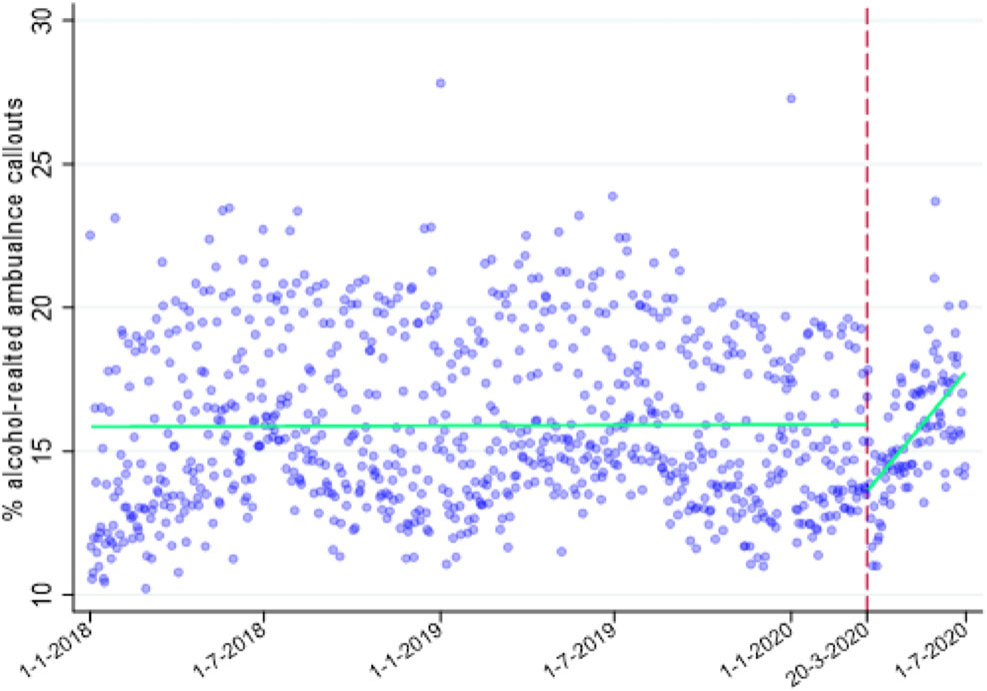
Alcohol‐related ambulance callouts as percentage of total callouts. Green solid lines are linear fitted trends over time before and after first day of lockdown, indicated by the dashed red line, when all hospitality venues (including pubs) were ordered to close.

Ambulance callouts of all causes from March to June 2020 decreased in number compared to the previous year, with falls of 1.1% in March, 12.3% in April, 11.5% in May and 10.9% in June (Figure 2, Table 3). Alcohol‐related ambulance callouts (Figure 3) fell much more sharply, driven largely by April figures, when they fell by 23%. In April 2020, the proportion of overall ambulance callouts related to alcohol was 14.5% or 2.2% lower than what it was in the same month in 2019 (16.7%); in relative terms, this was a 13% fall compared to the previous year. Both total callouts and alcohol‐related callouts fell by similar proportions in March, May and June 2020, considering callouts at all times of the week/day (Table 3). Prior to the pandemic, there was a consistently greater volume of alcohol‐related callouts at weekends compared to weekdays (Table 3), unlike other callouts (Figure 2). This difference disappeared in April 2020 when there were large drops in weekend alcohol‐related callouts and then reappeared gradually. Weekend (00:00 Friday to 23:59 Sunday) alcohol‐related callouts fell by 31.8% in April, whereas other weekend callouts fell by only 9.7%. The drop in alcohol‐related callouts was even more stark at weekend night‐times (20:00 Friday to 06:00 Saturday and 20:00 Saturday to 06:00 Sunday), which fell by 48.9% in April 2020 compared to a fall of 13.5% in other callout types (Table 3). After April, the proportion of alcohol related callouts gradually started to follow pre lockdown levels, but at weekends and weekend night‐times, alcohol‐related call‐outs were still substantially lower in June 2020 than in the previous year (Table 3).

**Table 3:** Ambulance callout counts and distribution in January–June 2019 and January–June 2020

	2019			2020			% Variation		
Month	Alcohol	Non‐alcohol	Overall	Alcohol	Non‐alcohol	Overall	Alcohol	Non‐alcohol	Overall
*Full week (Monday–Sunday)*									
January	7034	39 053	46 087	6887	38 133	45 020	−2.1%	−2.4%	−2.3%
February	6585	34 591	41 176	6354	35 076	41 430	−3.5%	1.4%	0.6%
March	7409	36 848	44 257	6423	37 337	43 760	−13.3%	1.3%	−1.1%
April	7298	36 263	43 561	5553	32 638	38 191	−23.9%	−10.0%	−12.3%
May	7451	37 463	44 914	6659	33 085	39 744	−10.6%	−11.7%	−11.5%
June	7622	36 957	44 579	6620	33 087	39 707	−13.1%	−10.5%	−10.9%
*Weekends (00.00 Friday–23.59 Sunday)*									
January	3056	15 074	18 130	3290	16 210	19 500	7.7%	7.5%	7.6%
February	3457	15 082	18 539	3404	16 045	19 449	−1.5%	6.4%	4.9%
March	4319	17 812	22 131	3164	15 684	18 848	−26.7%	−11.9%	−14.8%
April	3543	14 716	18 259	2416	13 289	15 705	−31.8%	−9.7%	−14.0%
May	3792	15 774	19 566	3400	16 070	19 470	−10.3%	1.9%	−0.5%
June	4244	17 481	21 725	2926	13 490	16 416	−31.1%	−22.8%	−24.4%
*Weekend nights (Friday 20.00 to Saturday 06.00 and Saturday 20.00 to Sunday 06.00)*									
January	1145	3543	4688	1263	3836	5099	10.3%	8.3%	8.8%
February	1380	3471	4851	1373	3878	5251	−0.5%	11.7%	8.2%
March	1706	4033	5739	953	3340	4293	−44.1%	−17.2%	−25.2%
April	1370	3368	4738	700	2913	3613	−48.9%	−13.5%	−23.7%
May	1517	3884	5401	1078	3465	4543	−28.9%	−10.8%	−15.9%
June	1659	4019	5678	991	3090	4081	−40.3%	−23.1%	−28.1%

The average number of alcohol related callouts per 100 000 residents in the month preceding lockdown (21 February–20 March 2020) was 4.06, but fell to 3.74 in the 3 months of lockdown (20 March–30 June 2020), reaching a low point in the first month (21 March–20 April 2020) of 3.36. Levels appeared to be returning to pre‐lockdown volumes from the end of May 2020 (3.93 alcohol related callouts per 100 000 residents in the week 22–28 May 2020).

The interrupted time series analysis found an association between lockdown and the proportion of callouts that were alcohol related. This proportion (alcohol‐related callouts as a percentage of total callouts) showed an absolute reduction of 2.14 percentage points (95% CI ‐3.54, ‐0.74; *P*=0.003). In relative terms, this corresponds to a 13% reduction in the proportion of callouts that were alcohol‐related compared to the period before the lockdown (1 January 2018–20 March 2020). This association was immediate and then lessened over time with a significant daily increase (post lockdown announcement) in the percentage of alcohol related callouts of 0.03% (95% CI 0.010, 0.05; *P* = 0.004). The analysis finds that the proportion of alcohol‐related call‐outs compared to total call‐outs came back to pre‐lockdown levels after approximately 2 months, at the end of May 2020 (Table 4 and Figure 3).

**Table 4:** Output of regression of interrupted time series on the percentage of alcohol related callouts

	Coefficient	*P* > |*z*|	95% confidence interval
Bank holiday	1.615	0.000	(1.172, 2.058)
Sat&Sunday	4.995	0.000	(4.508, 5.482)
Friday	1.735	0.000	(1.221, 2.249)
Trend	0.001	0.313	(−0.001, 0.004)
Lockdown	−2.142	0.003	(−3.542, −0.742)
Lockdown Trend	0.032	0.004	(0.010, 0.054)
January 1st	10.636	0.000	(0.117, 12.156)
Constant	13.274	0.000	(11.902, 14.645)

## Discussion

In most countries, the COVID‐19 pandemic and related public health measures profoundly changed aspects of life in ways that were largely unanticipated, including unprecedented changes to alcohol availability. Our data suggest at least four broad impacts with implications for alcohol harms and policy: relaxation of some aspects of licensing policy, significantly fewer alcohol‐related ambulance callouts initially followed by a resurgence, perceived increases in home drinking and diverse impacts on businesses.

### Implications for licensing policy

First, licensing stakeholders highlighted that restrictions on capacity and sales of alcohol indoors had led to an increase in applications from bars/pubs to serve alcohol in spaces outdoors and an increase in premises permitted to offer home delivery. When physical distancing remains a requirement, meaning reduced customer numbers indoors, extra outdoor space is unlikely to increase overall availability of alcohol, but it may increase risks of public disturbance via noise or other antisocial behaviour. It may be difficult to reverse outdoor licences granted during the pandemic, even when physical distancing is no longer required, suggesting a significant increase in overall capacity in those venues. A shift towards outdoor drinking renders alcohol consumption (and any related drunkenness) more visible, including to children and people in recovery from alcohol problems who may be passing by. The pandemic may have contributed to expansions in availability as licence applications received less public health scrutiny, but may have had some benefits in building relationships between stakeholders seen as important for facilitating successful public health engagement in licensing [44].

### Reductions in alcohol burden on ambulance services during lockdown

Second, qualitative data from the Scottish Ambulance Service indicate that the pandemic period, during which premises were either closed completely or not open late at night, was associated with a substantial reduction in demand for ambulances arising from alcohol consumption. The quantitative data shows that total alcohol‐related callouts fell even more than overall ambulance call‐outs in the first months of lockdown, but rebounded after 2 months. The reduction was particularly acute at weekend night‐times however, and call‐outs at these times remained lower than usual through to the third month after lockdown. Considering the reports of ambulance clinicians, it seems clear that reductions in call‐outs were linked to the closure of licensed premises and the night‐time economy (NTE), more than ‘stay at home’ measures introduced at the same time, even though these probably reduced socialising in people's homes. Exceptionally good weather in May 2020 may have mitigated the latter effect, perhaps explaining a rebound in weekend callouts that month even with premises still closed; that disappeared in June 2020. They reported clearly that, in normal times, many ambulance callouts are associated with people in or around pubs/bars/clubs in the NTE and that these were greatly reduced. The views expressed by paramedics are powerful and give pause for thought about whether business recovery post‐COVID has to mean a return to the ‘*mass intoxication*’ and ‘*battlefield environment*’ on city streets, which they described. As premises re‐open and especially in Scotland where the ambulance service is under intense pressure in 2021 [45], authorities should be looking to avoid this. There is surely an opportunity for politicians and clinicians to show leadership in pushing for better alcohol policies that protect frontline services. Effective multi‐faceted interventions already exist to reduce drunkenness [46] and violence [47] and could be more widely and consistently deployed, although are unlikely alone to be transformative.

### Transforming the night‐time economy to rely less on alcohol?

It is timely to consider whether economic prosperity in the NTE must rely on alcohol and whether there is a third way or ‘sweet spot’ approach via policies which transform and build the NTE to prioritise other forms of entertainment, food, music or more family‐friendly environments. The nature of such policies and their feasibility and acceptability to communities [48, 49] and trade stakeholders [50], plus their likely and actual effectiveness in balancing prosperity and reducing harms, requires further consideration and research. Strategic planning policies at local authority level [51], availability and promotion of no/very low alcohol products [52] and ‘place‐shaping’ in the licensing system, through which premises perceived as lower risk (restaurants, arts venues) are prioritised in licensing policy over others with a strong alcohol focus [48] may all have a role to play. Further work is underway to collate and assess the feasibility and acceptability of innovative initiatives in this space to inform policymaking [53].

### Increased home drinking during the pandemic

The third issue highlighted by both licensing stakeholders and ambulance clinicians, is that the pandemic shifted alcohol sales and drinking from the on‐ to off‐trade, exacerbating existing trends. Reportedly driven by increases in home delivery of alcohol coupled with closures of premises and ‘stay at home’ orders, interviewees' perceptions of increases in home drinking are supported by sales and survey data from other sources [7, 9, 54]. While the proportion of alcohol‐related callouts returned to that of pre‐lockdown periods shortly after May 2020, callouts were spread throughout the week; while the incidence at weekends remained persistently lower, especially at night. This suggests that after the first 2 months of lockdown, ambulance services may have already been seeing an increase in demand arising from home drinking. It is difficult to know what types of call‐outs would have generated these trends, however it could have arisen from a rise in outdoor get‐togethers/house parties, increased consumption in groups vulnerable to liver failure or acute harms or greater incidence of alcohol withdrawal. Research in Cardiff, Wales [55]) found a significant decrease in emergency department visits by people injured by violence, driven by a large reduction in visits due to violence outside the home. No significant increase in emergency department visits resulting from violence at home was noted. For injury outside the home, significant decreases were found in emergency department visits by female individuals younger than 18 years and by male individuals in all age groups, those injured with weapons and those in which the perpetrator was a stranger, acquaintance or security officer [55]. The relationship between emergency service utilisation and alcohol during the pandemic is likely to be highly context specific. It will also hugely depend on the pattern of restrictions in place as illustrated by findings from North America [26, 27] outlined earlier and evidence from South Africa finding reduced emergency attendances during total and partial alcohol sales bans [56].

### Overall implications of the pandemic for alcohol policy

Overall, it seems likely that the closure of licensed premises led to net reductions in the burden of alcohol‐related harm on emergency services, even with a shift to home drinking, but that this was relatively short‐lived. Total closures or prohibitions are neither practical nor desirable in a liberal society however and our data support the suggestions of others that an associated shift to home drinking may result in mid‐ and long‐term harms [5, 33]. In the longer term, the cheaper price of alcohol bought from off‐licence premises enables consumption of greater amounts of alcohol at home, raising the risk of conditions (cancer, hypertension, etc.) that would not arise during the follow‐up period in this study, nor be easily identified as alcohol‐related in ambulance/emergency department data. Interventions to raise the price of off‐trade alcohol (such as increases in or the introduction of minimum unit pricing) are likely the most effective available option to reduce shop‐bought alcohol consumption [57, 58]. Minimum unit pricing can also reduce the price differential between on and off trade premises, which may encourage people to drink in licensed premises; it may therefore support the hospitality sector, while protecting health [59]. Drinking (or drunkenness) at home is more visible to children than drinking by adults in bars/pubs where children are not permitted. Furthermore, for vulnerable subgroups, home drinking may be associated with exacerbations of domestic violence, isolation and alcohol dependence [4, 5]. As discussed by Reynolds and Wilkinson [3], the rise in home drinking illustrates a blind spot in licensing policy: systems largely designed to control ‘outlet density’ and maintain public order [60], have failed to adapt to address online sales or hidden harms associated with home drinking. The introduction of a ‘public health’ objective for alcohol licensing in Scotland perhaps signalled the intention to take a broader approach, but arguably without the requisite systems locally to fully realise hoped‐for benefits [44, 61]. There is now an opportunity to consider tighter controls on online sales/home delivery of alcohol, alongside pricing interventions as above, which would protect public health without impacting on (or perhaps supporting) the recovery of hospitality sectors post‐COVID.

### How the pandemic may be reshaping the on‐trade alcohol sector

A final issue raised by our data is that the pandemic is likely to have longer term impacts on the alcohol sector [62]. Smaller premises and nightclubs, even if permitted to open, have been particularly affected, as distancing requirements were often impractical. Independently‐owned businesses may be less likely to have the financial reserves to survive the hardship of lockdowns, despite government support and are reportedly being bought up by large pub companies [63]. Such chains may have greater lobbying power, which policymakers and other stakeholders in licensing will need to be equipped to handle. Furthermore, several larger pub companies also produce and supply alcohol for sale in shops and may therefore oppose measures to reduce home drinking, even if they might benefit bars. Independent bar owners and policy stakeholders should be mindful of these conflicting interests in trade associations that represent both on‐ and off‐trade interests.

### Strengths

This paper provides timely, relevant data to inform current debates around how to support businesses to recover from the COVID‐19 pandemic, while still protecting public health and health services. It makes pragmatic use of new data arising spontaneously in two studies not designed to focus on COVID‐19 and reports new analyses of data on ambulance call‐outs during the pandemic. Drawing together data from three studies allows for triangulation across the datasets and we found broad agreement on issues raised by the different sources, allowing us to draw questions and hypotheses from the findings.

### Limitations

There are however, some important limitations to bear in mind. The ExILEnS study was not focused on the pandemic and questions regarding the issues reported were not explored with all interviewees. Furthermore, relevant data were naturally only available from interviews conducted after the start of the first UK lockdown in March 2020, during which time only two public health stakeholders were interviewed. The views reported in the ExILEnS study are mostly those of others without a specific public health remit (but who work with/alongside public health)—a larger sample of public health stakeholders may broaden and strengthen findings relating to alcohol harm implications of the pandemic. In contrast, in the IMPAACT study, a strength is the varied sample of interviewees in terms of region, role and length of service and that questions about the pandemic were included in the topic guide. Both studies capture professional perspectives but there is also a need for in‐depth qualitative research to understand consumer experiences, including their view of possible long‐term outcomes of changes in alcohol consumption. Considering the quantitative ambulance callout data, while the algorithm used to identify alcohol‐related callouts has been found to be highly accurate [30], it may have over or underestimated alcohol‐related callouts. People may be less likely to call an ambulance for domestic incidents and it may be more difficult to identify alcohol as a factor in a callout to a home compared to callouts to licensed premises, which may be a source of bias. Nonetheless, the drop in callouts is unlikely to be explained solely by these factors, is triangulated by the qualitative reports of ambulance clinicians and mirrors the findings regarding emergency department visits from a fairly similar context in Cardiff, Wales [55]).

## Conclusion

Drawing on new data from three studies, we report potential impacts of the COVID‐19 pandemic that have implications for public health and alco hol policy. Licensing stakeholders reported the liberalisation of licensing laws that may increase availability of alcohol and the possible reshaping of the on‐trade sector as different premises types and businesses are more or less negatively affected by the pandemic. There were large short‐term reductions in alcohol‐related ambulance callouts while public health measures closed or severely restricted on‐trade premises, especially at weekend night‐times and reports from both licensing and ambulance interviewees of rises in home drinking with potential long‐term consequences. Paramedics gave stark reports about the volume of late night alcohol‐related callouts prior to the pandemic. We argue that these reports should give pause for thought about the wisdom of a return to ‘normal’ in the night‐time economy and tentatively draw on prior literature to suggest possible approaches to both reduce harm and support business recovery. At a time when policymakers are reluctant to be seen to hurt already suffering hospitality sectors, but also wish to protect health services, it is going to be vital for politicians, advocates and lobbyists alike to find win–win’ policies that can do both.

## Conflict of Interest

The authors have no conflicts of interest.
